# Can computed tomography be a primary tool for COVID-19 detection? Evidence appraisal through meta-analysis

**DOI:** 10.1186/s13054-020-02908-4

**Published:** 2020-05-06

**Authors:** Edward Pei-Chuan Huang, Chih-Wei Sung, Chi-Hsin Chen, Cheng-Yi Fan, Pei-Chun Lai, Yen-Ta Huang

**Affiliations:** 1 Department of Emergency Medicine, National Taiwan University Medical College and Hospital, Taipei, Taiwan; 2 Department of Emergency Medicine, National Taiwan University Hospital Hsin-Chu Branch, Hsinchu, Taiwan; 3 Department of Medicine Education, National Taiwan University Hospital, Taipei, Taiwan; 4 Evidence-based Medicine Center, Department of Medical Education, Hualien Tzu Chi Hospital, Buddhist Tzu Chi Medical Foundation, 707, Sec. 3, Chung-Yang Rd, Hualien, 970 Taiwan; 5 Department of Pediatrics, Hualien Tzu Chi Hospital, Buddhist Tzu Chi Medical Foundation, 707, Sec. 3, Chung-Yang Rd, Hualien, 970 Taiwan; 6 School of Medicine, Tzu Chi University, Hualien, Taiwan; 7 Division of Experimental Surgery, Department of Surgery, Hualien Tzu Chi Hospital, Buddhist Tzu Chi Medical Foundation, 707, Sec. 3, Chung-Yang Rd, Hualien, 970 Taiwan; 8 Surgical Intensive Care Unit, Department of Surgery, Hualien Tzu Chi Hospital, Buddhist Tzu Chi Medical Foundation, 707, Sec. 3, Chung-Yang Rd, Hualien, 970 Taiwan; 9 Department of Pharmacology, Tzu Chi University, Hualien, Taiwan

**Keywords:** Computed tomography, COVID-19, Meta-analysis, Likelihood ratio, Sensitivity, Specificity

The World Health Organization (WHO) has officially declared the pandemic of coronavirus disease 2019 (COVID-19) on March 11, 2020 [1]. The current pandemic COVID-19 causes suspicious cases flocking into hospitals. The detection of COVID-19 by traditional reverse-transcription diagnostic polymerase chain reaction (RT-PCR) tests is time-consuming and depends on the reliability of laboratory techniques. Several PCR-based rapid tests have been recently approved and only require less than 30 min. Chest computed tomography (CT) has been suggested as an alternative and reliable tool for the detection of COVID-19 in symptomatic patients in China [2]. However, the American College of Radiology recommended against the use of CT as a first-line test to diagnose COVID-19 on March 11, 2020 [3]. To validate this recommendation, we performed a systematic review with meta-analysis to evaluate the diagnostic value of chest CT in COVID-19.

Two investigators independently searched with the term of “novel coronavirus” or “coronavirus disease 2019” or “COVID-19” or “SARS-CoV-2” combined with “computed tomography” or “CT” on PubMed, Web of Science, Embase, Cochrane Library, and China Academic Journals Full-text Database (CJFD) till March 13, 2020. Studies were excluded due to duplication, irrelevant topics, case report(s) or series, availability of only the abstract, and insufficient data. Two investigators independently extracted data for pooled estimates of sensitivity, specificity, and positive and negative likelihood ratio [LR(+) and LR(−)] with 95% confidence intervals (CIs) calculated by midas command in Stata 15 (StataCorp LLC., College Station, TX, USA). Heterogeneity across studies was examined using *I*^2^. Fagan’s Nomogram plot analysis was performed to compare the pre-test probability, the LR, and the post-test probability.

Only 4 studies screened from 372 relevant articles were eligible [2, 4–6]. A total of 1286 patients in China were screened for COVID-19 using both RT-PCR and chest CT. The pooled sensitivity and specificity of chest CT were 0.95 (95% CI, 0.93–0.97) and 0.09 (95% CI, 0.02–0.34), respectively, using RT-PCR as the reference method (Fig. 1). The pooled LR (+) and LR (−) of chest CT were as low as 1.10 (95% CI, 0.90–1.20) and 0.49 (95% CI, 0.10–2.33), respectively. We further used Fagan’s Nomogram to calculate the post-test probability of diagnosed COVID-19 by chest CT (Fig. 2). Our analysis revealed that, regardless the levels of pre-test probabilities (25, 50, and 75%), the post-test probabilities were only slightly changed.

**Fig. 1:**
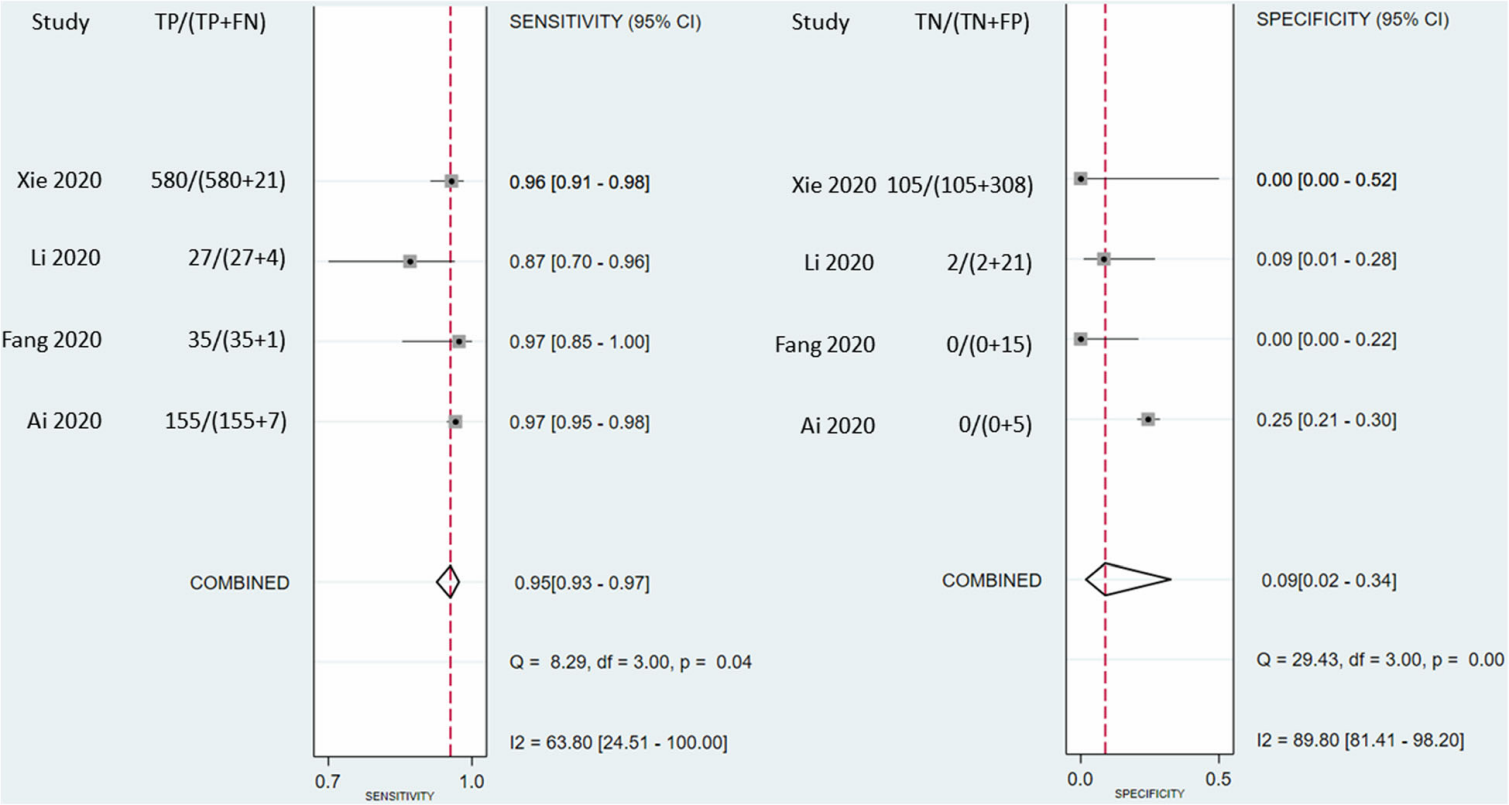
Results of meta-analysis for the evaluation of the diagnostic value of chest CT in COVID-19. Study-specific and mean of sensitivity and specificity are presented in the Forest plots. TF, true positive; FN, false negative; TN, true negative; FP, false positive; CI, confidence interval

**Fig. 2:**
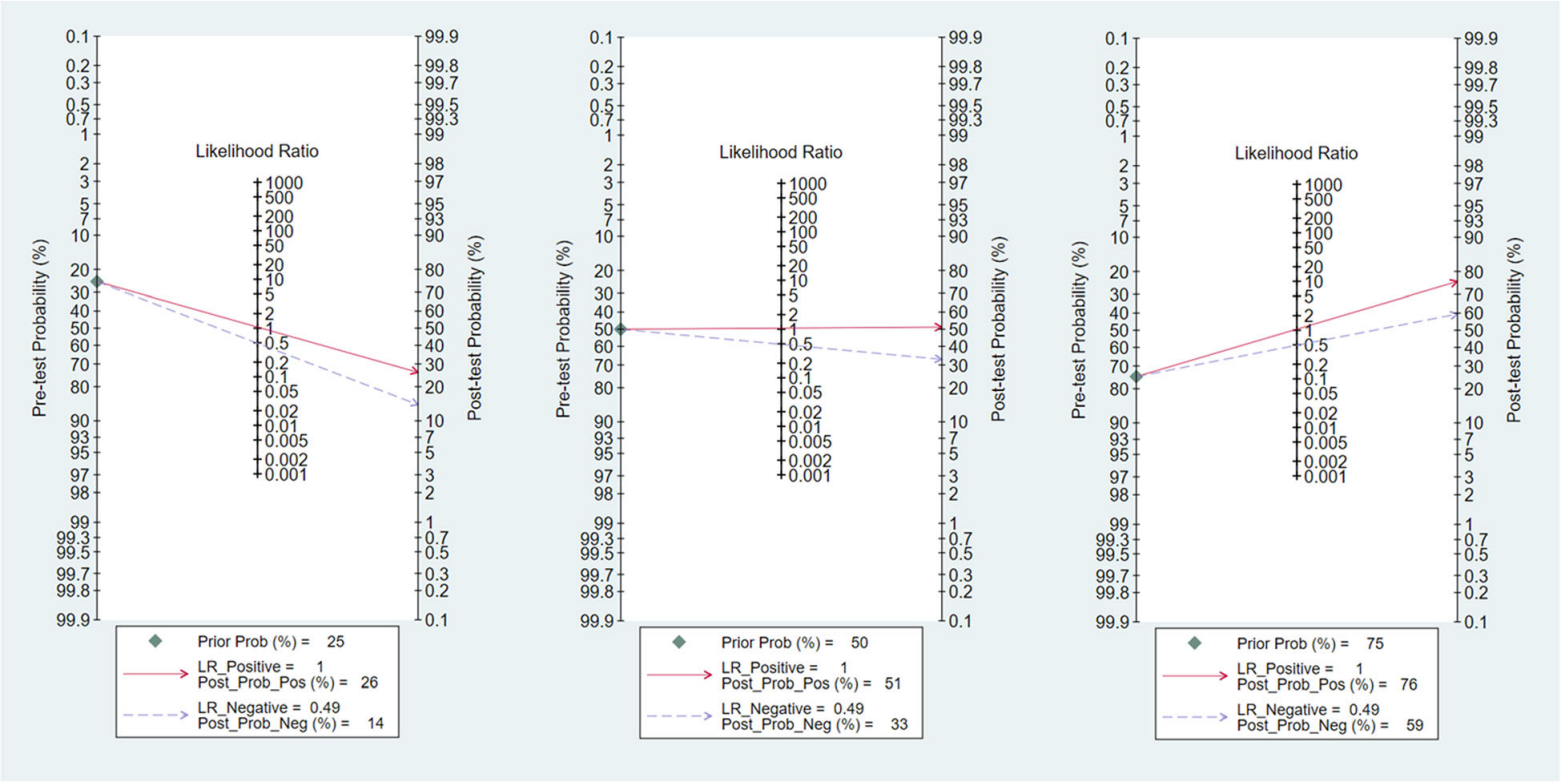
Evaluating the clinical utility of chest CT for COVID-19 detection by Fagan’s Nomogram plot. LR, likelihood ratio; prob, probability; pos, positive; neg, negative

Our results indicate a high sensitivity of chest CT for the detection of COVID-19. However, our results regarding low levels of specificity and likelihood ratio did not support the routine use of chest CT for COVID-19 screening in suspicious patients. Our results from Fagan’s Nomogram analyses suggested very little diagnostic value of using chest CT as the primary tool for COVID-19. One shortcoming of using chest CT is that patients are exposed to unnecessary radiation. The other shortcoming is that CT-scanning may increase the risk of nosocomial infection due to potential contamination of the environment.

Some limitations of our results should be mentioned. For example, whether radiologists were blind to other clinical data when interpreted CT or whether samples were adequately collected may influence the pooled results. Besides, we observed a high heterogeneity of both sensitivity and specificity as well as a wide range of specificity. The certainty of evidence, if graded, may be very low.

In conclusion, our pooled meta-analytic results of high sensitivity but poor specificity limit the routine use of chest CT as a primary tool for COVID-19 detection. Chest CT should only be arranged for individuals with certain clinical features in conjunction with RT-PCR tests. Further rigorous studies are required to find further refinements of our findings.

## Data Availability

Not applicable.
